# Editorial: Z-curve Applications in Genome Analysis

**DOI:** 10.2174/138920291502140421153952

**Published:** 2014-04

**Authors:** Chun-Ting Zhang

It is my pleasure to be a guest editor for the special issue on *Z*-curve applications in genome analysis. We are now living in the genomic era and facing an avalanche of sequencing information; two decades ago, however, not a single genome sequence of free-living organisms was available. The progress in DNA sequencing technology, in terms of both speed and cost, has been remarkable.

The discovery of the double helix structure of DNA by James Watson and Francis Crick in 1953 marked the birth of molecular biology, and gave rise to a series of landmark findings and inventions that made DNA sequencing possible. In 1995, Venter and colleagues published the first genome sequence of a free-living organism, the bacterium *Haemophilus influenzae*, which has a genome of 1,830,137 bases. The Human Genome Project, which aimed to sequence the three billion bases in the human genome, was completed in 2003. As of October 2013, the number of sequenced bases deposited in GenBank has reached 1.55 × 10^11^ from nearly 260,000 formally described species, in addition to 5.35 × 10^11^ bases from whole genome shotgun projects. Technology that has the capacity to sequence the entire human genome within hours for under $1000, such as the nanopore detection system, is on the horizon. The amount and speed in the accumulation of sequencing data are unprecedented in human history.

DNA is a fascinating molecule. The basic form of DNA is extremely simple, as it is composed of only four kinds of chemical bases, adenine, guanine, cytosine and thymine, but the information that it conveys is astonishing; DNA encodes the genetic instructions used in the development and functions of almost all organisms from bacteria to humans. The avalanche of DNA sequence information presents a tremendous challenge on how to decode what is hidden in the DNA sequence to obtain biologically meaningful knowledge.

I commenced the research on DNA sequence analysis in the 1980s. In physics, algebraic and geometric methods are often jointly used to address a particular problem. With a background in theoretical physics, one of my aims was to develop a geometrical method, or a curve, that constitutes a one-to-one correspondence with the DNA sequence, so that the study of DNA could be shifted to the study of the curve. Considerable symmetry appears to exist in the DNA molecule, which, in turn, can be represented as a DNA group. Based on this group theory, the one-to-one correspondence to a DNA sequence reveals a three-dimensional curve with a zigzag shape, hence the name *Z*-curve. In the past two decades, the *Z*-curve method has found applications in a wide range of areas in genome analysis, including the identification of protein-coding genes, promoters, translation start sites, replication origins, isochores and horizontally-transferred genomic islands in various genomes. 

The identification of protein-coding genes in a genome represents one of the most classical bioinformatics endeavors. A bacterial genome can have a large number of open reading frames (ORFs), while only a small subset is *bona fide* protein-coding, and the goal is to identify this subset. According to the *Z*-curve method, ORF nucleotide compositions are represented uniquely by mapping points in a high-dimensional space, and protein-coding ORFs and non-coding ones usually locate in distinct regions. Discriminant algorithms or other classifiers can then be applied to distinguish the two kinds of mapping points. This method, although straightforward, has been proven to be effective and accurate for gene identification in bacterial, archaeal and eukaryotic genomes. In this special issue, Drs. Guo, Lin and Chen review *Z*-curve applications in identification of protein-coding genes in bacterial and archaeal genomes.

DNA replication, as the basis of biological inheritance, is critical for all organisms. DNA replication starts at a specific location, termed the origin of replication (*oriC*). Bacterial genomes have a single *oriC*, whereas archaeal genomes can have either single or multiple *oriC*s. The RY, MK, AT and GC disparity curves (different *Z*-curve components) have been successfully used for identification of *oriC*s in many bacterial and archaeal genomes. Dr. Gao reviews *Z*-curve applications in *oriC* identification, as well as the development of *oriC* finding software and databases. 

The genomic island is the part of a genome that is acquired through horizontal transfer. Genomic islands can code for many functions, such as those for symbiosis or pathogenesis, and can play a role in bacterial drug resistance. Horizontal gene transfer is increasingly being recognized as a universal event throughout bacterial evolution. Based on the *Z*-curve method, a windowless method for GC content calculation, the GC profile, has been proposed. In this issue, Drs. Zhang, Ou and colleagues review the use of the GC profile in identifying horizontally-transferred genomic islands in bacterial genomes, and in identifying homogenous domains in eukaryotic genomes.

In this special issue, I will deduce the *Z*-curve method from the aspect of group theory, as a foundation for the *Z*-curve algorithms. Three included reviews focus on the identification of protein-coding genes, replication origins and genomic islands. Over the past two decades, significant advancements have been achieved in genome analysis using the *Z*-curve method, and further advances are anticipated. Twenty years ago, before the publication of the first bacterial genome sequence, it was not practical even for the most visionary scientists to anticipate what we know now about genomes, such as the high degree of eukaryotic transcriptome complexity, single-cell genomics, synthetic genomes, and the Neanderthal genome. Thus, it is probably a safe bet that our present genome understanding in another twenty years will seem obsolete. Incorporation of the *Z*-curve method in future research on genome analysis, therefore, may happen in ways that we cannot currently imagine.

